# Kinesin KIFC3 is essential for microtubule stability and cytokinesis in oocyte meiosis

**DOI:** 10.1186/s12964-024-01589-8

**Published:** 2024-03-29

**Authors:** Jia-Qian Ju, Hao-Lin Zhang, Yue Wang, Lin-Lin Hu, Shao-Chen Sun

**Affiliations:** 1 College of Animal Science and Technology, Nanjing Agricultural University, Nanjing, 210095 China; 2 Key Laboratory of Research on Clinical Molecular Diagnosis for High Incidence Diseases in Western Guangxi, Reproductive Medicine, Guangxi Medical and Health Key Discipline Construction Project, Affiliated Hospital of Youjiang Medical University for Nationalities, Baise, China

**Keywords:** KIFC3, Oocyte, Meiosis, Spindle, Cytokinesis

## Abstract

KIFC3 is a member of Kinesin-14 family motor proteins, which play a variety of roles such as centrosome cohesion, cytokinesis, vesicles transportation and cell proliferation in mitosis. Here, we investigated the functional roles of KIFC3 in meiosis. Our findings demonstrated that KIFC3 exhibited expression and localization at centromeres during metaphase I, followed by translocation to the midbody at telophase I throughout mouse oocyte meiosis. Disruption of KIFC3 activity resulted in defective polar body extrusion. We observed aberrant meiotic spindles and misaligned chromosomes, accompanied by the loss of kinetochore-microtubule attachment, which might be due to the failed recruitment of BubR1/Bub3. Coimmunoprecipitation data revealed that KIFC3 plays a crucial role in maintaining the acetylated tubulin level mediated by Sirt2, thereby influencing microtubule stability. Additionally, our findings demonstrated an interaction between KIFC3 and PRC1 in regulating midbody formation during telophase I, which is involved in cytokinesis regulation. Collectively, these results underscore the essential contribution of KIFC3 to spindle assembly and cytokinesis during mouse oocyte meiosis.

**Supplementary Information:**

The online version contains supplementary material available at 10.1186/s12964-024-01589-8.

## Introduction

Mammalian oocyte meiosis is a distinctive and precise process that leads to the formation of haploid germ cells [1]. The organization of the bipolar spindle at the equatorial plate during metaphase I (MI) represents an important event following germinal vesicle breakdown (GVBD). Subsequently, homologous chromosomes segregate and move towards the spindle poles in anaphase I (AI) and telophase I (T1), accompanied by migration towards the cortex and formation of an actin cap. Finally, polar body extrusion occurs along with completion of cytokinesis [2]. Therefore, the meiotic spindle assembly and the cytokinesis are two key events during the maturation of mammalian oocytes.

Unlike mitosis, mouse oocyte meiosis orchestrates the arrangement of microtubules into a bipolar spindle in the absence of centrosomes [3]. Acentriolar microtubule-organizing centers (aMTOCs) act as a major site for microtubule nucleation, replacing centrosomes. Initially dispersed throughout the cytoplasm, aMTOCs subsequently aggregate the chromosomes to reassemble into a bipolar spindle, indicating that the self-organization of these structures drives acentriolar spindle assembly [4]. Notably, key components such as pericentrin, Cep192 and γ-tubulin are encompassed within aMTOCs [5]. Besides, the Ran-mediated pathway for microtubule nucleation plays an essential role in facilitating spindle assembly [6]. While post-translational modification of tubulin, especially tubulin acetylation [7], is also an important factor affecting microtubule stability and spindle formation [8], since tubulin acetylation is reported to alter microtubule structure [9]. Sirt2 is reported to involve into oocyte microtubule deacetylation and spindle assembly, which is crucial for oocyte development [10].

In a manner akin to mitosis, meiosis is tightly regulated to ensure accurate chromosome segregation through the involvement of the anaphase promoting complex (APC/C) and the spindle assembly checkpoint (SAC) [11]. Typical checkpoint kinases in meiosis include Bub1, Mps1, Mad2, BubR1 (Mad3), Bub3, and Aurora B and C [12]. The successful attachment of microtubules to kinetochores represents a critical event for SAC activation [13]. BubR1 and Bub3 have been shown to monitor kinetochore-microtubule attachments in oocytes [14, 15]. Once chromosomes align and kinetochores attach to microtubules, the SAC becomes inactive leading to chromosome separation and entry into anaphase. The central spindle or central region is composed of tightly interconnected microtubules that extend from the poles to the center of the spindle, forming antiparallel microbundle structures between the separated chromosomes [16]. Then, the contracted cleavage groove and the microtubules in the medial region form a special structure called the mestome at the telophase. The formation of an intermediate region is crucial for the separation of chromosomes and cytokinesis [17]. PRC1, a crucial element situated at the spindle midzone, plays a significant role in regulating spindle orientation throughout mitosis [18]. Additionally, previous studies have highlighted the essentiality of PRC1 in midzone formation and cytokinesis during mouse oocyte development. These processes are tightly controlled by KIF4A [19].

Kinesin motor protein family comprises a total of 45 kinesin proteins, which are further categorized into 14 distinct subclasses. Kinesins play pivotal roles in diverse cellular processes including chromosome segregation, spindle formation, microtubule dynamics, cytokinesis, and progression through both mitotic and meiotic cell cycles [20]. Unlike other kinesins, the members of the kinesin 14 subfamily possess a motor domain at their C-terminal region instead of the N-terminal region, enabling them to move along microtubules in an opposite direction to the minus-end. In mammalian cells, this family comprises four proteins (KIF25, KIFC1, KIFC2 and KIFC3) [21]. According to reports, KIF25 has been found to play a crucial role in preserving chromosome alignment, thereby ensuring the stable orientation of the spindle during mitosis initiation [22]. KIFC1 has been demonstrated to have a significant impact on the processes of mitosis and meiosis [23, 24]. The involvement of KIFC3 in the regulation of cellular proliferation and mitosis has been reported in ESCC cell lines [25]. KIFC3 has also been reports to control the epithelial-to-mesenchymal transition by modulating the PI3K/AKT/mTOR signaling pathway [26, 27]. Furthermore, KIFC3 is deactivated by NEver in mitosis-related Kinase 2 (NEK2), which facilitates assembly of the EG5-driven bipolar spindle. The role of KIFC3 in ensuring persistent centrosome cohesion during mitosis and its impact on chromosome segregation [28]. It has also been reported that the distinctive motor-dependent localization of KIFC3 at the central bridge facilitates its interaction with microtubules, thereby enhancing efficient progression through cytokinesis in meiosis [29]. And KIFC3, a microtubule minus end–directed motor, is also implicated in the apical transport of Triton-insoluble membranes associated with annexin XIIIb [30].

While previous studies have provided insights into the functions of KIFC3 in mitotic cells, its role in meiosis remains unexplored. This study aimed to investigated the role of KIFC3 in mouse oocyte meiosis by perturbing its activity. Our findings demonstrated that KIFC3 localized to centromeres during metaphase I and translocated to the midbody at telophase I. These findings highlight the unique contribution of KIFC3 in participating in spindle assembly and cytokinesis during the maturation process of mouse oocytes.

## Materials and methods

### Oocyte collection and in vitro culture

The experiments were conducted on 4-week-old female ICR mice, which had been approved by the Animal Research Committee of Nanjing Agricultural University, China, and performed in accordance with institutional guidelines. The female ICR mice were euthanized using cervical dislocation. Oocytes from 4-week-old ICR mice were cultured in M2 medium under conditions of 37 °C liquid paraffin oil and a mixture of 5% CO2 in air. All necessary reagents and media were procured from Sigma-Aldrich unless specified otherwise.

### Microinjection of KIFC3 morpholino, siRNA and antibody

The microinjections were conducted within a time frame of 30 minutes using an inverted microscope (Olympus IX71; Olympus, Japan) and a micromanipulator and microinjector system (Eppendorf AG, Hamburg, Germany). Morpholino (Morpholino oligo: 5’ to 3’ GGCTCAGTAACCTCTTCTGGGTGCC), Anti-KIFC3 antibody (0.5 mg/ml in PBS) and siRNA (Santa Cruz, sc-146480) were injected into cytoplasm at GV oocytes. In all experiments, a microinjection volume ranging from 5 to 10 pl per oocyte was utilized. To ensure reliable results, each experiment was conducted with three independent replicate groups, with approximately 100 oocytes injected in each group. As a control, an equivalent amount of IgG diluted in PBS solution was also injected. The cultured oocytes were subsequently used for further investigations.

### **Antibodies and chemicals**

The rabbit polyclonal anti-KIFC3 antibody (10125-2-AP) and rabbit polyclonal anti-Sirt2 (19655-1-AP) were purchased from Proteintech (Rosemont, IL, USA). The anti-α-tubulin-FITC antibody (F-2168), Hoechst 33342 (B2261), and mouse monoclonal anti-acetylated tubulin antibody (T7451) were obtained from Sigma (St. Louis, MO, USA). The mouse monoclonal anti-PRC1 antibody (sc-376983) was acquired from Santa Cruz Biotechnology (Santa Cruz, CA, USA). The rabbit monoclonal anti-Bub3 antibody (ab133699), rabbit monoclonal anti-gamma-tubulin antibody (ab179503), and sheep polyclonal anti-BubR1 antibody (ab28193) were purchased from Abcam (Cambridge, UK). Anti-β-actin (4970) and the rabbit monoclonal p-Aurora A/B/C antibody (D13A11) were obtained from Cell Signaling Technology (Danvers, MA, USA). Rabbit polyclonal antibodies against H3S10ph (42704) were sourced from Gene Tex (Irvine, CA, USA). Human anti-centromere CREST antibodies (15–234) were purchased from Antibodies Incorporated (Davis, CA, USA). AlexaFluor 594 goat anti-mouse antibodies (A11005) and AlexaFluor 488 goat anti-rabbit antibodies (A11008) were acquired from Invitrogen (Carlsbad, CA, USA). Conjugated goat antirabbit/mouse IgG (H + L) antibodies were procured from CWBIO (Beijing, China).

### Immunofluorescence staining and confocal microscopy

Oocytes at specific stages were immersed in a solution of PBS containing 4% paraformaldehyde for a period of 30 min. Following that, they underwent permeabilization using PBS with 0.5% Triton X-100 for a duration of 20 min and were subsequently blocked in PBS supplemented with 1% bovine serum albumin at room temperature for an hour. Afterward, oocytes were exposed to primary antibodies after being washed three times using PBS containing 0.1% Tween 20 and 0.01% Triton X-100. Then, secondary antibodies (Alexa Fluor-conjugated 488 and 594 goat anti-rabbit/mouse IgG) diluted at a ratio of 1:200 were applied to the oocytes. Finally, Hoechst 33,342 was used to stain the chromosomes for a period of time lasting approximately 20 min. The resulting images were observed utilizing a laser-scanning confocal fluorescent microscope (Zeiss LSM700 META, Jena, Germany). The primary antibodies used for immunoblotting were: anti-KIFC3 (1:100), anti-α-tubulin-FITC antibody (1:500), anti-Crest (1:100), anti-gamma-tubulin (1:200), anti-acetylated tubulin antibody (1:500), anti-H3S10ph (1:100), anti-p-Aurora A/B/C (1:100), anti-Sirt2 (1:100), anti-PRC1(1:100).

### Cold treatment

The oocytes at the MI stage were subjected to a brief freezing process at 4 °C for a duration of 10 min in order to induce depolymerization of the unstable microtubules. Subsequently, Anti-CREST and anti-α-tubulin antibody were employed to label and examine the enduring microtubules and the connection between kinetochore and microtubules.

### Nocodazole treatments

10 mg/ml Nocodazole (Sigma, M1404) was introduced into M2 medium at a proportion of 1:1000, resulting in a final concentration of 10 µg/ml. MI oocytes were subjected to a 10-minute treatment with Nocodazole in M2 medium at 37 °C.

### Western blotting

Using NuPAGE LDS sample buffer to lyse cells at 100 °C for 10 min. The proteins underwent separation through electrophoresis using sodium dodecyl sulfate-polyacrylamide gel at a voltage of 160 V for a duration of 80 min, and then membrane transferred (Millipore, Billerica, MA, USA) at 20 V for 100 min and blocked using western Rapid Transfer Buffer (P0572, Beyotime, Shanghai, China) for 20 min. Followed by incubation overnight at 4 °C with anti-KIFC3 (1:500), anti-PRC1 (1:500), anti-SIRT2 (1:500) after washing in TBST 3 times (10 min each), then was incubated with secondary antibody at room temperature for 1 h after washing in TBST 5 times (5 min each). Finally, the membranes were exposed using the ECL Plus Western Blotting Detection System Tanon-3900 (Tanon, China).

### Co-immunoprecipitation

10 ovaries were lysed in an appropriate lysis buffer on ice, followed by centrifugation at 12,000 g for 15 min to obtain the supernatant. The anti-KIFC3 antibody was conjugated with Dynabeads Protein G (ThermoFisher Scientific) and incubated at room temperature for 3–4 h. Subsequently, the mixture was subjected to three washes using a lysis buffer to remove any unbound proteins. The antibody-conjugated Dynabeads were then exposed to the supernatant overnight at 4 °C, resulting in formation of immune complexes that were subsequently analyzed via western blotting for protein interaction analysis. In the control group, IgG served as the control antibody while Input consisted of 10 µl of lysate without any antibodies present.

### Chromosome spread

The oocytes were subjected to a 1-minute treatment with Tyrode solution for zona pellucida removal. Subsequently, they were exposed to 1% sodium citrate, transferred onto a glass slide, and fixed using several drops of methanol-acetic acid mixture (in a ratio of 3:1). After air drying and nuclear staining, the chromosomes were visualized using a confocal microscope.

### Statistical analysis

The experiments were performed with biological replicates conducted a minimum of three times. Statistical analysis was carried out using GraphPad Prism 7 software (GraphPad, San Diego, CA) employing the T-test method. The results are presented as mean ± SEM. A significance level of *P* < 0.05 was considered to determine statistical significance.

## Result

### KIFC3 is essential for mouse oocyte meiotic maturation

**Fig. 1:**
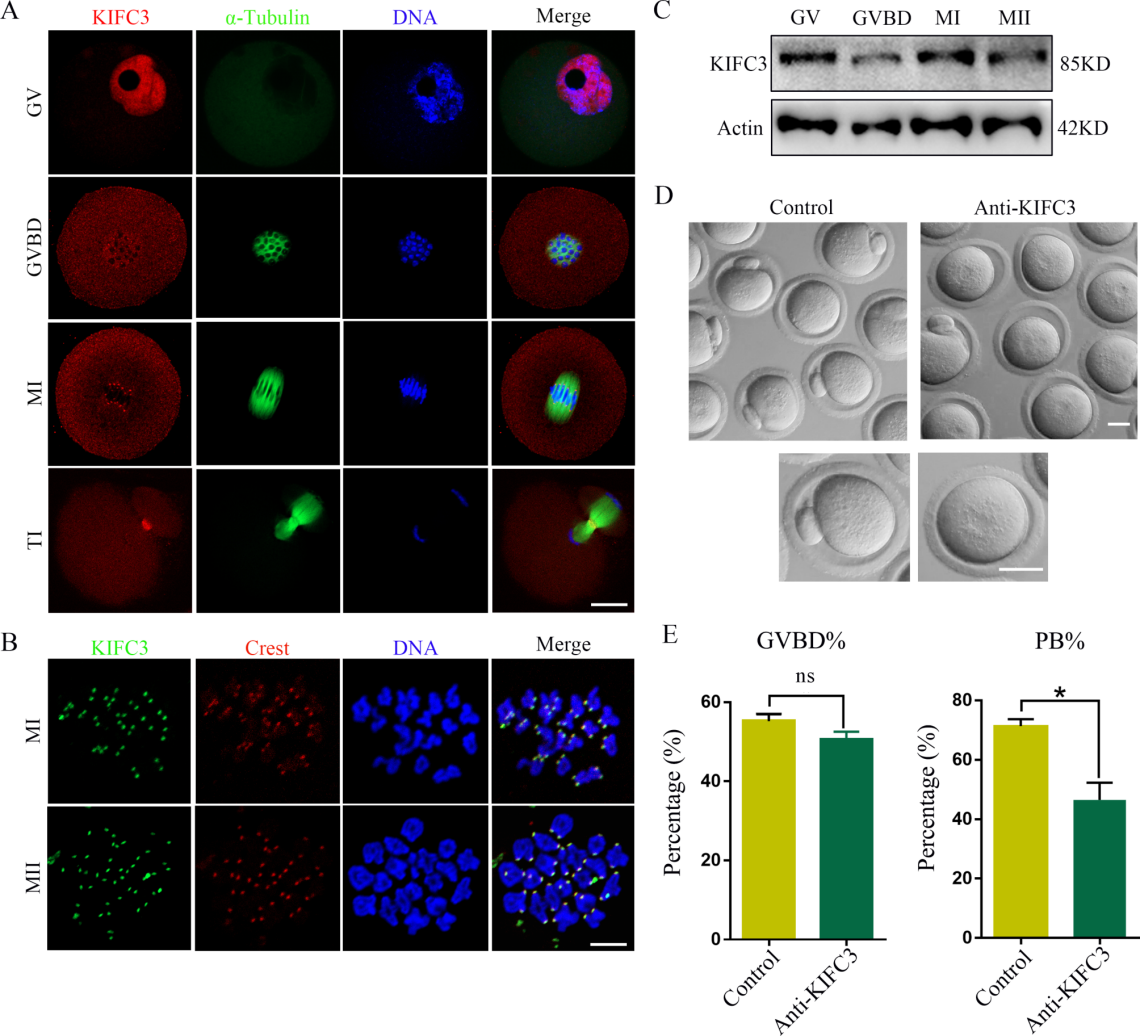
KIFC3 is essential for mouse oocyte meiotic maturation (**A**) Oocytes were immunolabeled with anti-α-tubulin (green) and anti-KIFC3 antibody (red), and Hoechst 33,342 was used to label DNA (blue). Bar = 20 μm. (**B**) Co-localization of KifC3 with Crest at MI and MII stages. Oocytes cultured to 8 h (MI) and 12 h (MII) were stained for KIFC3 (green), Crest (red), and DNA (blue). Bar = 10 μm. (**C**) Immunoblotting assays for the expression level of KIFC3 at different stages (GV, GVBD, MI, and MII) during mouse oocyte maturation. (**D**) The typical picture of control oocytes and KIFC3-antibody injection oocytes. Bar = 50 μm. (**E**) Quantitative analysis of GVBD rate and PB1 extrusion rate in control and KIFC3-antibody injection oocytes. Graph shows means ± sem of results obtained in 3 independent experiments. *P < 0.05

Mouse oocytes were cultured for 0, 4, 8, 10 and 12 h, corresponding to the GV, GVBD, MI, TI and MII phases respectively, in order to investigate the localization and expression of KIFC3 during meiosis. As shown in Fig. 1A, during the GV phase, KIFC3 was mainly distributed in the nucleus; after GVBD, KIFC3 accumulated in the vicinity of the condensed chromosomes; at the MI stage, KIFC3 accumulated strongly at the centromeres; KIFC3 was observed to localize in the midbody at telophase I; During the MII stage, a distinct concentration of KIFC3 was identified surrounding the centromeres instead. The localization pattern observed in KIFC3 differs from the previously reported pattern during mitosis. To conduct a more comprehensive analysis of the precise localization of KIFC3 on chromosomes, we utilized chromosome spreading and performed immunofluorescent staining using CREST. Notably, at both the MI stage and MII stage, KIFC3 accumulated brightly in the vicinity of CREST (Fig. 1B). The expression of KIFC3 during meiotic maturation was subsequently investigated. Our findings demonstrated that KIFC3 exhibited consistent expression throughout all stages of meiosis in mouse oocytes (Fig. 1C).

We subsequently investigated the roles of KIFC3 in mouse oocytes by employing siRNA and morpholino injection to suppress KIFC3 expression. Unfortunately, we found that KIFC3 protein expression have no significant differences between control and siRNAs/morpholino injection oocytes, and the rate of polar body extrusion also had not significant differences (Fig. S1). Next, we used KIFC3 antibody injection to interfere with KIFC3 protein activity and the findings demonstrated a significant impact on the extrusion of the first polar body after the KIFC3 antibody injection (Fig. 1D). The suppression of KIFC3 did not result in a significant alteration in the rate of GVBD [control 55.4 ± 1.67%, *n* = 90, versus KIFC3-antibody 50.67 ± 1.86%, *n* = 95, not significant] (Fig. 1E). However, the suppression of KIFC3 resulted in a significant decrease in the occurrence of polar body extrusion compared to control oocytes. [control 71.36 ± 2.35%, *n* = 90, versus KIFC3-antibody 46.52 ± 5.78%, *n* = 95, *P* < 0.05] (Fig. 1E). These findings suggested that KIFC3 is crucial for the process of meiotic maturation in mouse oocytes.

### KIFC3 regulates meiotic spindle formation in mouse oocyte meiosis

**Fig. 2:**
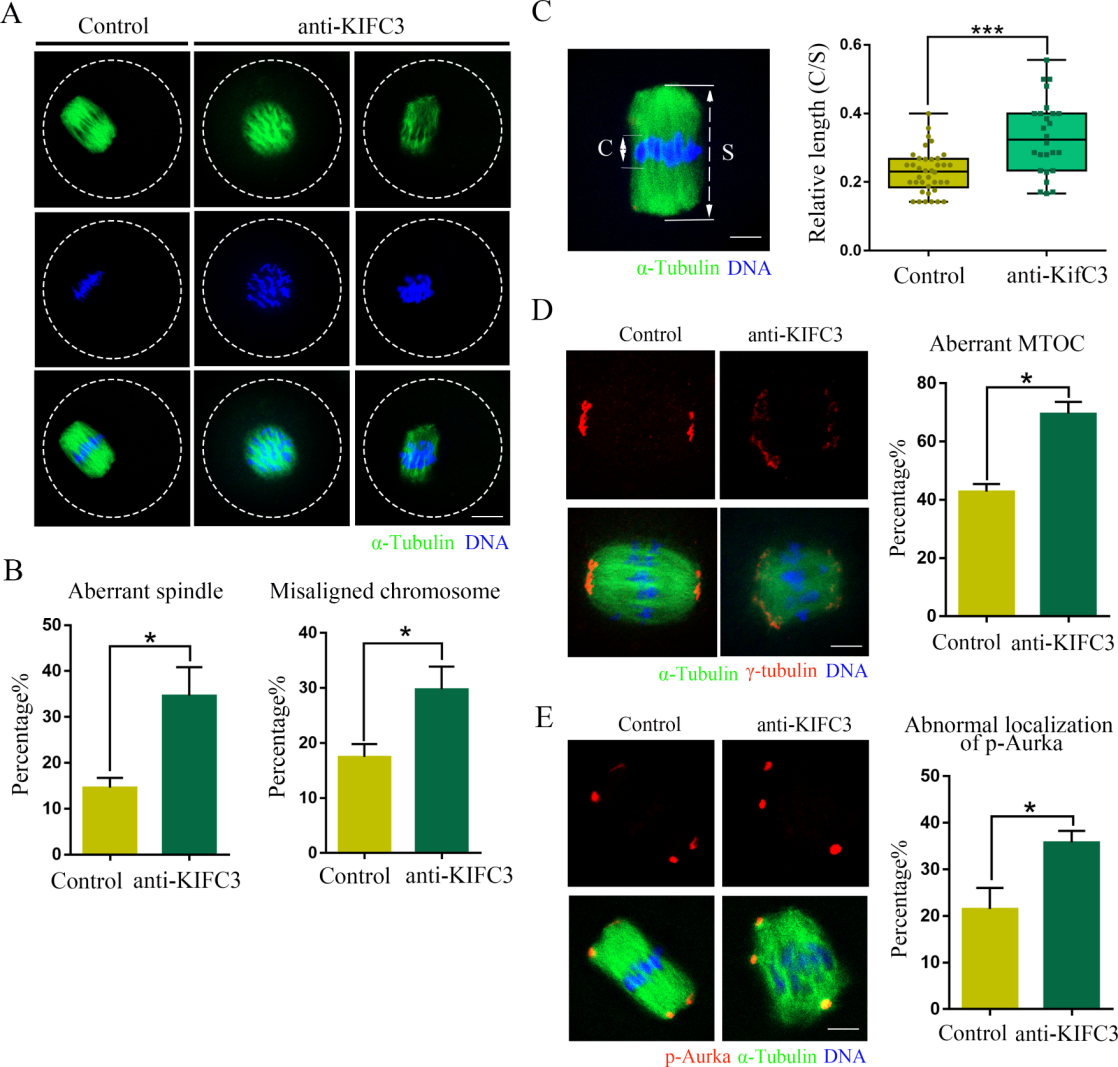
KIFC3 regulates spindle formation and chromosome alignment in mouse oocyte meiosis (**A**) Control and KIFC3-antibody injection oocytes at the MI stage were stained with anti-α-tubulin (green) and counterstained with Hoechst 33,342 to visualize the chromosomes (blue). Bar = 20 μm. (**B**) The rate of abnormal spindle morphology and misaligned chromosome after KIFC3-antibody injection at the MI stage. Graph shows means ± sem of results obtained in 3 independent experiments. *P < 0.05. (**C**) Thickness of the spindle middle plate. C indicates the maximal span of chromosomes; S indicates the maximal spindle length. Bar = 10 μm. The scattergram shows the C: S ratios for control and KIFC3-antibody injection oocytes at the MI stage. Graph shows means ± sem of results obtained in 3 independent experiments. ***P < 0.001. (**D**) Immunofluorescence analysis of γ-tubulin (red) and α-tubulin (green) in control and KIFC3-antibody injection oocytes. Bar = 10 μm. The histogram shows the percentages of control and KIFC3-antibody injection oocytes with abnormal γ-tubulin. Graph shows means ± sem of results obtained in 3 independent experiments. *P < 0.05. (**E**) Immunofluorescence analysis of p-Aurka (red) and α-tubulin (green) in control and KIFC3-antibody injection oocytes. Bar = 10 μm. The histogram shows the percentages of control and KIFC3-antibody injection oocytes with abnormal p-Aurka. Graph shows means ± sem of results obtained in 3 independent experiments. *P < 0.05

We subsequently examined spindle morphology following KIFC3 inactivation and observed a significant disruption in spindle assembly. Control group oocytes exhibited typical barrel-shaped spindles with accurate chromosome alignment at the MI stage. In contrast, treated group oocytes displayed pronounced aberrant spindles and severe misalignment of chromosomes. These aberrant spindles were characterized by reduced size and diminished microtubule signals. (Fig. 2A). The statistical analysis revealed a significantly higher percentage of abnormal spindles in the KIFC3 inactivation group compared to the control group [control 14.62 ± 2.12%, *n* = 65, versus KIFC3-antibody 34.63 ± 6.23%, *n* = 75, *P* < 0.05] (Fig. 2B). Meanwhile, the percentage of misaligned chromosomes in the KIFC3 inactivity group was significantly higher than that in the control group [control 17.47 ± 2.34%, *n* = 65, versus KIFC3-antibody 29.79 ± 4.11%, *n* = 75, *P* < 0.05] (Fig. 2B). To quantitatively evaluate the phenotype, we conducted measurements the width of the spindle midzone, which represents the spatial extent occupied by condensed chromosomes (Fig. 2C). The middle plate was significantly broader in KIFC3 inactivity oocytes than in control oocytes [control 0.23 ± 0.01, *n* = 38, versus KIFC3-antibody 0.33 ± 0.02, *n* = 26, *P* < 0.05] (Fig. 2C). The MTOC protein γ-tubulin is important for spindle formation during mouse oocyte meiosis. We explored the effect of KIFC3 inactivity on the localization of γ-tubulin. The γ-tubulin failed to concentrate at spindle poles in a high percentage of KIFC3 inactivity oocytes [control 42.81 ± 2.60%, *n* = 32, versus KIFC3-antibody 69.57 ± 4.09%, *n* = 38, *P* < 0.05] (Fig. 2D). Moreover, we also examined the localization of the key spindle assembly protein Aurka and the results showed that the Aurka also failed to locate at the spindle poles in KIFC3 inactivity oocytes [control 21.5 ± 4.50%, *n* = 36, versus KIFC3-antibody 35.78 ± 2.49%, *n* = 28, *P* < 0.05] (Fig. 2E).

### KIFC3 regulates kinetochore-microtubule attachment in mouse oocytes

**Fig. 3:**
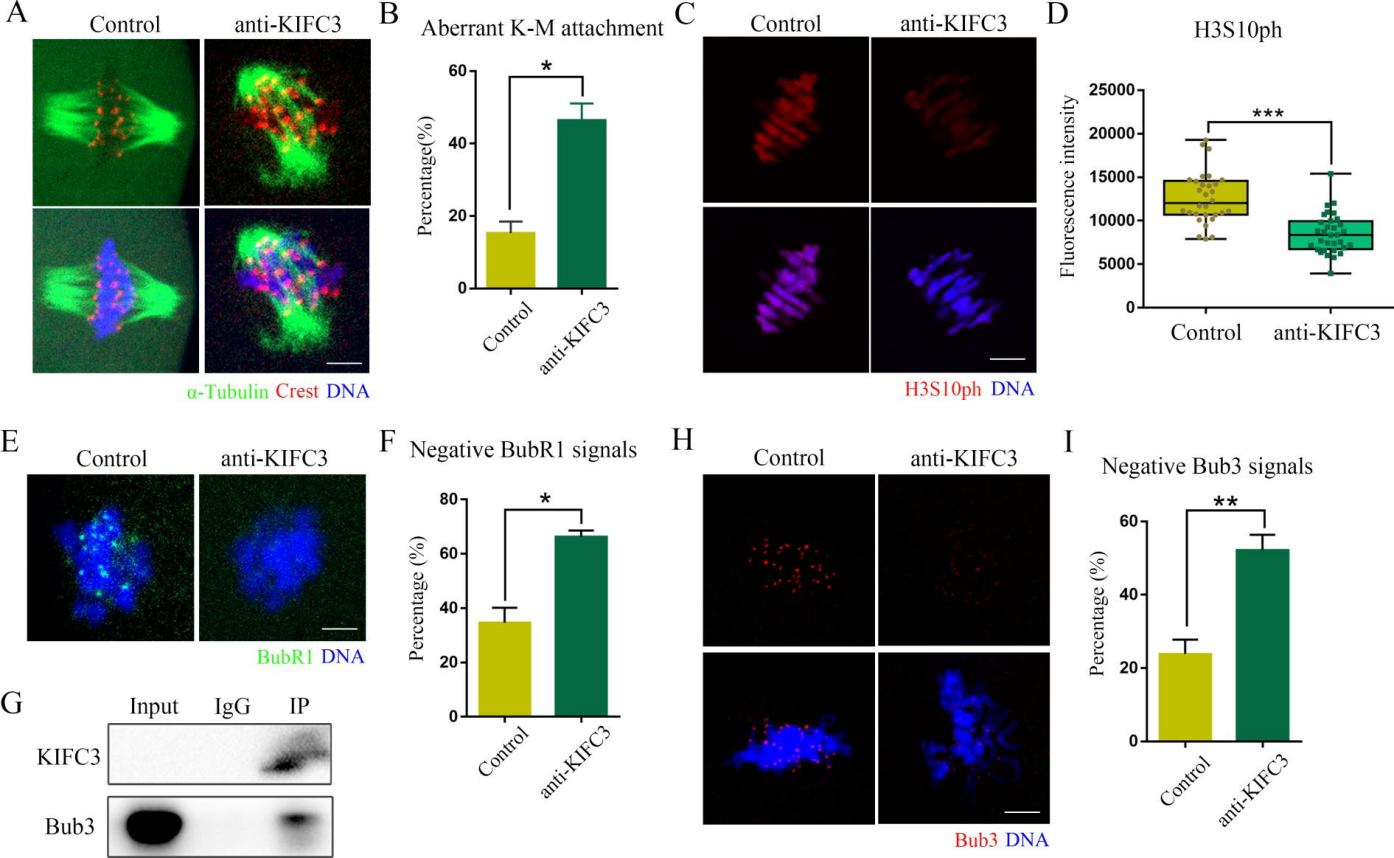
KIFC3 regulates kinetochore-microtubule attachment in mouse oocytes (**A**) Control and KIFC3-antibody injection oocytes at the MI stage were stained with anti-α-tubulin (green), anti-CREST (red) and counterstained with Hoechst 33342 to visualize the chromosomes (blue). Bar = 10 μm. (**B**) The rate of abnormal kinetochore-microtubule attachment after KIFC3-antibody injection at the MI stage. Graph shows means ± sem of results obtained in 3 independent experiments. *P < 0.05. (**C**) Control and KIFC3-antibody injection oocytes at the MI stage were stained with anti-H3S10ph (red) and counterstained with Hoechst 33342 to visualize the chromosomes (blue). Bar = 5 μm. (**D**) The scattergram shows the relative fluorescence intensity of H3S10ph signals in control and KIFC3-antibody injection oocytes. Graph shows means ± sem of results obtained in 3 independent experiments. ***P < 0.001. (**E**) Control and KIFC3-antibody injection oocytes at the MI stage were stained with anti-BubR1 (green) and counterstained with Hoechst 33342 to visualize the chromosomes (blue). Bar = 5 μm. (**F**) The histogram shows the percentages of control and KIFC3-antibody injection oocytes with abnormal BubR1. Graph shows means ± sem of results obtained in 3 independent experiments. *P < 0.05. (**G**) Co-IP was performed with an anti-KIFC3 antibody. The immunoblots of protein precipitates were probed with an anti-Bub3 antibody. (**H**) Control and KIFC3-antibody injection oocytes at the MI stage were stained with anti-Bub3 (red) and counterstained with Hoechst 33342 to visualize the chromosomes (blue). Bar = 5 μm. (**I**) The histogram shows the percentages of control and KIFC3-antibody injection oocytes with abnormal Bub3. Graph shows means ± sem of results obtained in 3 independent experiments. **P < 0.01

Based on the observed localization pattern in MI oocytes, our hypothesis posits that KIFC3 may play a regulatory role in kinetochore-microtubule attachment during meiosis. To evaluate the stability of these attachments, we subjected them to cold treatment, inducing depolymerization of any unstable microtubules not bound to kinetochores. In control oocytes, we observed precise and proper attachment between kinetochores and microtubules. However, in KIFC3 inactive oocytes, there was a failure to establish appropriate kinetochore-microtubule attachment (Fig. 3A). The incidence of kinetochore-microtubule defects was higher in KIFC3 inactivity oocytes than in control oocytes [control 15.26 ± 3.23%, *n* = 39, versus KIFC3-antibody 46.53 ± 4.54%, *n* = 42, *P* < 0.05] (Fig. 3B). Histone H3 phosphorylated at Ser-10 (H3S10ph) is crucial for chromosome condensation. We detected the levels of H3S10ph between control and KIFC3 inactivity oocytes by immunofluorescence staining (Fig. 3C). The fluorescence intensity of H3S10ph signals was significantly lower in KIFC3 inactivity oocytes than in control oocytes [control 12,617 ± 540.6, *n* = 30, versus KIFC3-antibody 8509 ± 403.4, *n* = 32, *P* < 0.05] (Fig. 3D). In addition, SAC protein BubR1 and Bub3 are essential for kinetochore-microtubule attachment in oocytes and promoted chromosome alignment. Thus, we observed the localization of BubR1 on chromosomes in MI oocytes (Fig. 3E), and the results showed that a large proportion of KIFC3 inactivated oocytes had no detectable BubR1 signal on chromosomes [control 34.66 ± 5.52%, *n* = 35, versus KIFC3-antibody 66.39 ± 2.17%, *n* = 27, *P* < 0.05] (Fig. 3F). Moreover, the co-IP results showed that Bub3 interacted with KIFC3 (Fig. 3G), while the location of Bub3 also showed the similar results with BubR1 [control 23.89 ± 3.89%, *n* = 31, versus KIFC3-antibody 52.15 ± 4.23%, *n* = 35, *P* < 0.01] (Fig. 3H, I). Therefore, we speculated that KIFC3 could recruit SAC protein Bub3 to the kinetochore to regulate kinetochore-microtubule attachment.

### KIFC3 regulates spindle microtubule stability in mouse oocytes meiosis

**Fig. 4:**
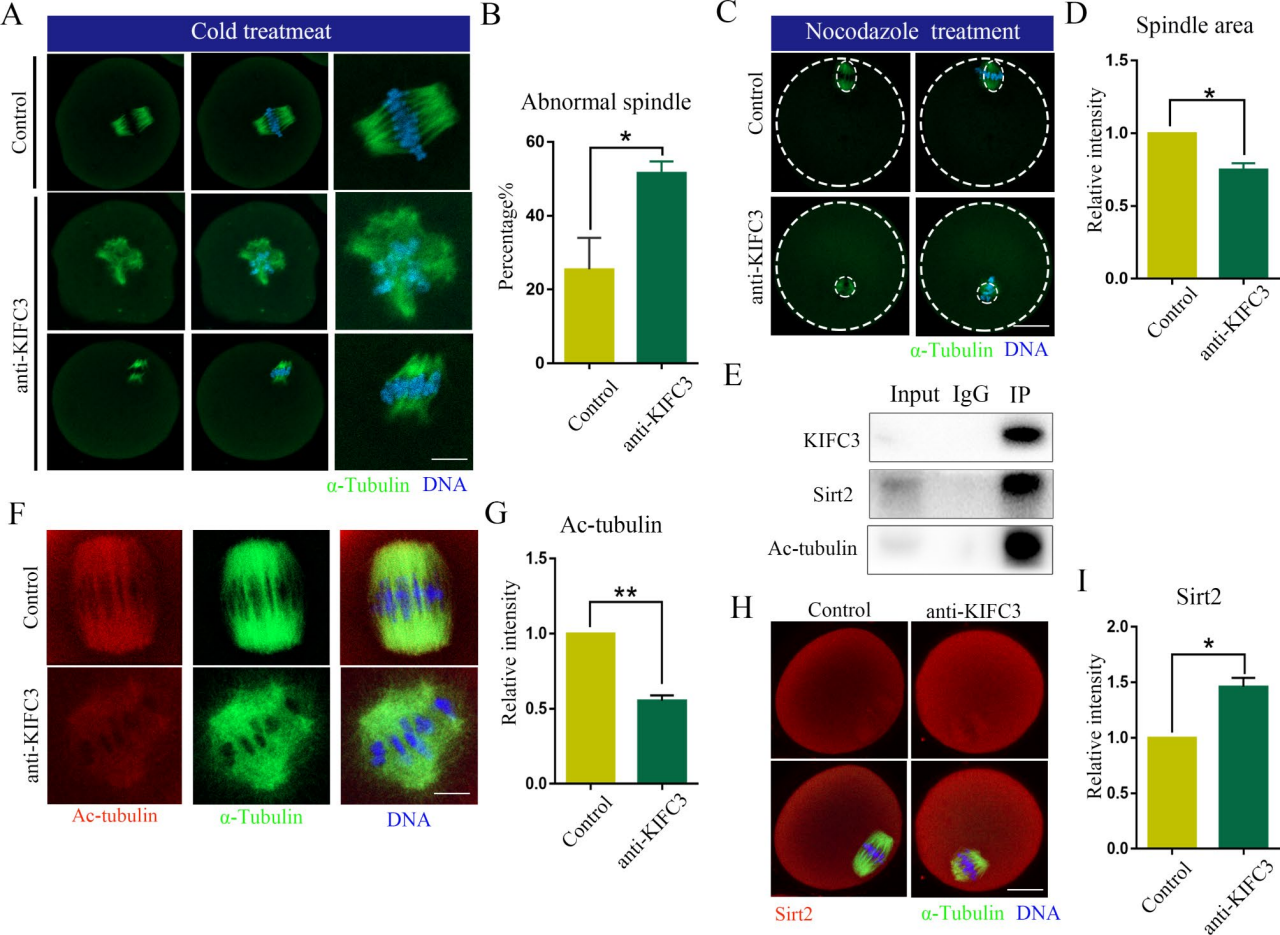
KIFC3 regulates stability of spindle microtubules (**A**) Control and KIFC3-antibody injection oocytes after cold treatment at the MI stage were stained with anti-α-tubulin (green) and counterstained with Hoechst 33342 to visualize the chromosomes (blue). Bar = 20 μm. (**B**) The rate of abnormal spindle morphology after cold treatment of KIFC3 antibody injection and control oocytes at the MI stage. Graph shows means ± sem of results obtained in 3 independent experiments. *P < 0.05. (**C**) Control and KIFC3-antibody injection oocytes after Nocodazole treatment at the MI stage were stained with anti-α-tubulin (green) and counterstained with Hoechst 33342 to visualize the chromosomes (blue). Bar = 20 μm. (**D**) The relative area of spindle after nocodazole treatment of KIFC3 antibody injection and control oocytes at the MI stage. Graph shows means ± sem of results obtained in 3 independent experiments. *P < 0.05. (**E**) Co-IP was performed with an anti-KIFC3 antibody. The immunoblots of protein precipitates were probed with anti-Sitr2 and anti- acetylated tubulin antibody. (**F**) Immunofluorescence analysis of acetylated tubulin (red) and α-tubulin (green) in control and KIFC3 antibody injection oocytes. Bar = 10 μm. (**G**) The histogram shows the relative fluorescence intensity of acetylated tubulin signals in control and KIFC3 antibody injection oocytes. Graph shows means ± sem of results obtained in 3 independent experiments. **P < 0.01. (**H**) Immunofluorescence analysis of Sirt2 (red) and α-tubulin (green) in control and KIFC3 antibody injection oocytes. Bar = 20 μm. (**I**) The histogram shows the relative fluorescence intensity of Sirt2 signals in control and KIFC3 antibody injection oocytes. Graph shows means ± sem of results obtained in 3 independent experiments. *P < 0.05

To explore the underlying causes of spindle defects after KIFC3 inactivation, we evaluated microtubule stability and the levels of endogenous acetylated tubulin. First, we observed the microtubule stability of oocytes treated with cold treatment and nocodazole, and the results showed that the degree of microtubule depolymerization of oocytes deactivated with KIFC3 after cold treatment was higher than that of the control group [control 25.53 ± 8.50%, *n* = 35, versus KIFC3-antibody 51.62 ± 3.12%, *n* = 36, *P* < 0.05] (Fig. 4A, B); Moreover, the spindle of KIFC3 inactivity oocytes was also significantly smaller than that of the control group treated with nocodazole [control 1 ± 0, *n* = 31, versus KIFC3-antibody 0.75 ± 0.05, *n* = 29, *P* < 0.05] (Fig. 4C, D). Microtubule acetylation was known to be important for microtubule assembly and microtubule stability. We detected the relationship between KIFC3, Ac-tubulin and deacetylase Sirt2, and the co-IP results showed that they were correlated (Fig. 4E). Ac-tubulin expression was much lower in KIFC3 inactivated oocytes than in control oocytes [control 1 ± 0, *n* = 39, versus KIFC3-antibody 0.5 ± 0.04, *n* = 36, *P* < 0.05] (Fig. 4F, G). Besides, the levels of Sirt2 fluorescence intensity in KIFC3 inactivated oocytes were significantly higher than those in the control group [control 1 ± 0, *n* = 39, versus KIFC3-antibody 1.46 ± 0.08, *n* = 41, *P* < 0.05] (Fig. 4H, I). Therefore, we believed that KIFC3 regulated the acetylation level of microtubules by interacting with sirt2, thus affecting the stability of microtubules.

### KIFC3 regulates midbody formation and cytokinesis in mouse oocytes

**Fig. 5:**
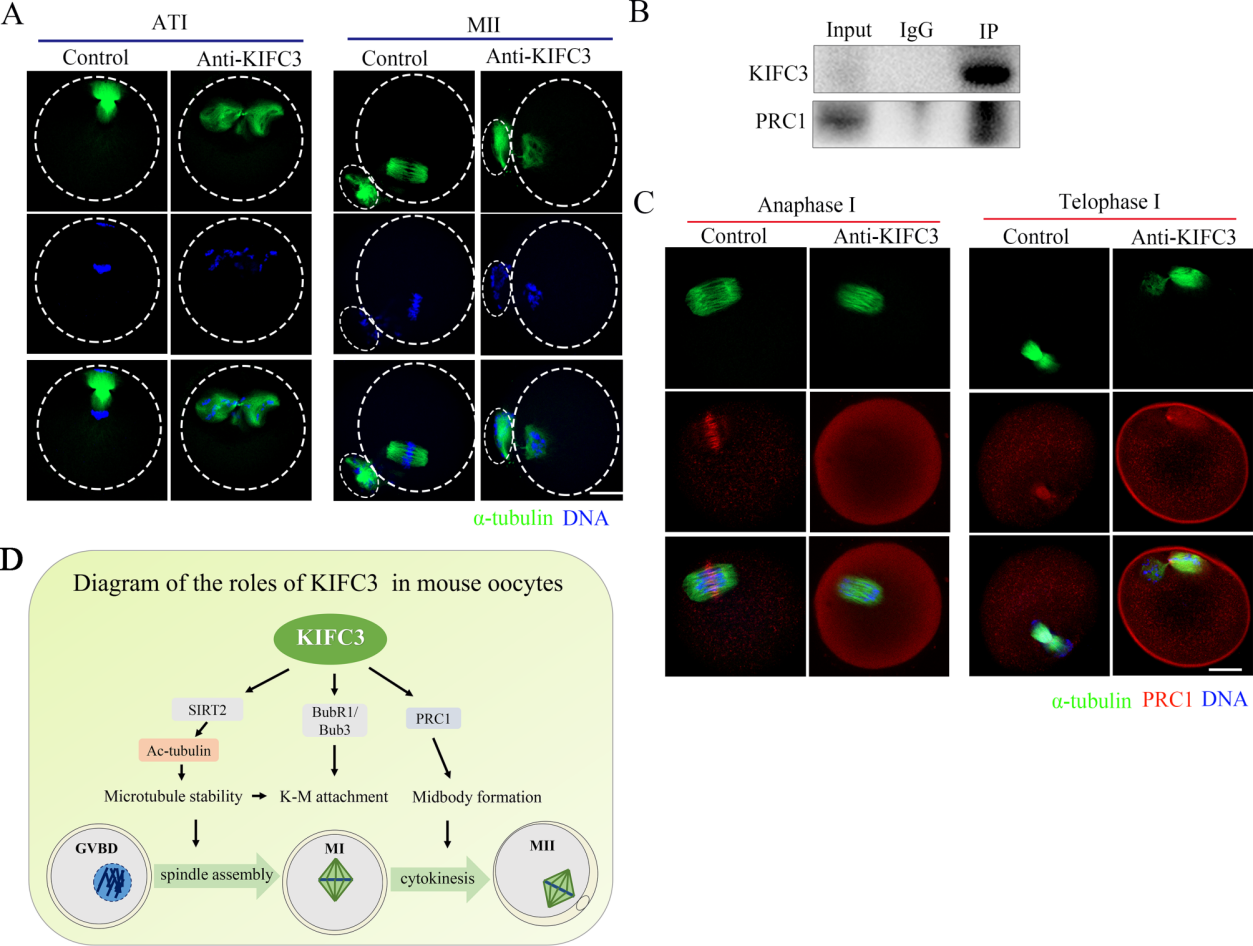
KIFC3 affects midbody formation and cytokinesis in mouse oocytes (**A**) Spindle morphology at the ATI stage and MII stage oocytes. Green, α-tubulin; blue, DNA. Repeat the experiment 4 times. Bar = 20 μm. (**B**) Co-IP was performed with an anti-KIFC3 antibody. The immunoblots of protein precipitates were probed with anti-PRC1 antibody. (**C**) Control and KIFC3-antibody injection oocytes at the ATI stage were stained with anti-α-tubulin (green), anti-PRC1 (red) and counterstained with Hoechst 33342 to visualize the chromosomes (blue). Repeat the experiment 3 times. Bar = 20 μm. (**D**) Diagram of the roles of KIFC3 during mouse oocyte meiotic maturation. KIFC3 regulated tubulin acetylation to stabilize the microtubules for spindle assembly and kinetochore-microtubules attachment, and KIFC3 also interacted with PRC1 for cytokinesis during mouse oocyte meiosis

Due to the KIFC3 localization at the midbody at telophase I, we obtained oocytes that were treated with KIFC3 antibody and subjected to a 10–12-hour culture period in order to investigate the development of spindle/midzone structures during TI and MII stages of oocyte maturation. Oocytes lacking KIFC3 showed aberrant telophase I spindle with no midzone formed. Conversely, the majority of control oocytes displayed normal morphology in the midzone region. Following 12 h of culture, oocytes injected with KIFC3 antibodies at the MII stage demonstrated disrupted spindle structures, while control oocytes maintained a normal spindle (Fig. 5A). Based on this finding, we performed Co-IP and the findings indicated that KIFC3 interacts with PRC1 (Fig. 5B). We observed the localization of PRC1 in the anaphase I and telophase I oocytes, and the results showed that in KIFC3 inactivated oocytes, the localization of PRC1 in the midzone was missing (Fig. 5C). Therefore, we speculated that KIFC3 played a role in the formation of the midbody and cytokinesis in mouse oocytes through its impact on the localization of PRC1.

## Discussion

KIFC3, a member of the kinesin superfamily proteins (KIFs), is widely acknowledged for its pivotal role in facilitating cellular cargo transportation during mitosis [26]. However, the understanding of the requirements for oocyte meiotic maturation by KIFC3 remains limited. In this study, our objective was to investigate the functional roles of KIFC3 in mouse oocyte maturation. To disrupt KIFC3 activity, we administered an injection of a specific antibody targeting KIFC3 and observed its crucial involvement in maintaining spindle microtubule stability, facilitating kinetochore-microtubule attachment, promoting spindle formation, and ensuring proper cytokinesis during meiosis I in oocytes. These findings provided valuable insights into the significant roles and potential regulatory mechanisms associated with KIFC3 during oocytes meiosis (Fig. 5D).

In mitosis, KIFC3 is concentrated at spindle poles; while we observed a different distribution pattern of KIFC3 in mouse oocytes. We showed We observed the localization of KIFC3 at the centromere during the MI stage, followed by its translocation to the midbody during telophase I. Depletion of KIFC3 resulted in aberrant spindle formation and misalignment of chromosomes. These findings underscore the indispensable role of KIFC3 in both spindle assembly and chromosome congression. In the process of mitosis, KIFC3 functions as a kinesin protein that regulates the initiation of assembly for the mitotic spindle and plays a crucial role in maintaining centrosome cohesion by exerting significant force [28]. KIFC3 in neurons has a more structural role by working together with minus-end binding protein CAMSAP2 to organize dendritic microtubules [31]. During oogenesis, the degeneration of centrioles leads to the assembly of the spindle via chromatin-mediated microtubule nucleation and self-organization of multiple cytoplasmic MTOCs in mammalian oocyte meiosis [32, 33]. Thus, in mouse oocytes KIFC3 may have an unclassical role in the absence of typical centrioles. γ-tubulin, a member of MTOC, as the key protein responsible for microtubule nucleation [34]. In addition, Aurka co-localizes with MTOCs throughout meiosis and is required for recruitment of γ-tubulin [35], and Aurka deletion results in meiotic spindle defects, which is similar to the phenotypes observed in our study [36]. These findings further confirmed that KIFC3 inactivity affected γ-tubulin and Aurka-based meiotic spindle assembly.

Furthermore, considering that KIFC3 was aggregates on centromeres during the MI phase, we further examined kinetochore-microtubule attachment, a progress which guarantees chromosome alignment. Our results showed that KIFC3 was essential for kinetochore-microtubule attachment in oocytes. SAC protein involves in regulating kinetochore-microtubule attachment. For example, The exposure to low temperatures in BubR1-depleted oocytes during meiosis I resulted in the disruption of spindle microtubules, indicating that BubR1 plays a role in monitoring kinetochore-microtubule attachments [14]. Additionally, Bub3 influenced the connections between microtubules and kinetochores during the transition from metaphase to anaphase [15]. Our results showed that Bub3 interacted with KIFC3, and the loss of KIFC3 activity resulted in the failure of recruitment of Bub3/BubR1 to the kinetochores. Therefore, we speculated that KIFC3 interacts with SAC protein regulate kinetochore-microtubule attachment. Furthermore, the critical role of acetylated tubulin in maintaining microtubule stability has been established [9, 37]. Our findings demonstrated that KIFC3 regulated the levels of tubulin acetylation, thereby influencing the stability of microtubules in mouse oocytes. Subsequently, we aimed to investigate the underlying mechanism by which KIFC3 modulates tubulin acetylation. Tubulin deacetylation was mainly regulated by the deacetylases such as SIRT2 [7]. It has been reported that overexpression of Sirt2 leads to a reduction in the level of α-tubulin acetylation in aged mouse oocytes [38], which aligns with our observed results indicating an increase in SIRT2 levels following KIFC3 antibody injection, subsequently resulting in decreased α-tubulin acetylation. Co-IP results indicated the potential association between KIFC3 and SIRT2. As a motor protein, KIFC3 may transport SIRT2 to the termination for the deacetylation of tubulin protein. When loss of KIFC3 activity interrupt the transport, it may induce the aberrant accumulation of SIRT2. Whether SIRT2 is the direct cargo of KIFC3 or it exists regulatory factors associated with KIFC3 and SIRT2 still needs more studies to clarify.

KIFC3 plays a significant role in formation of the central bridge morphology as cells transition from telophase to cytokinesis. The unique motor-dependent localization of KIFC3 at the central bridge promotes efficient progress through cytokinesis in mitosis [29]. Our data showed that KIFC3 was localized at the midbody at telophase I in oocytes. During the ATI stage of mouse oocyte meiosis, KIF4A predominantly accumulated in the midbody [39]. And KIF4A also plays a crucial role in facilitating the movement of PRC1 along microtubules, thereby contributing to the precise organization of the spindle midzone and regulation of cytokinesis [19]. Therefore, we hypothesized that KIFC3 was association with PRC1 to regulate cytokinesis. Our data demonstrated that KIFC3 and PRC1 were interacted, and the inactivation of KIFC3 affected PRC1 to enrich at the midzone. Hence, it is hypothesized that the potential involvement of KIFC3 in facilitating the positioning of PRC1 at the spindle midzone could play a role in governing cytokinesis regulation in mouse oocytes.

In brief, our findings suggested that the regulation of tubulin acetylation by KIFC3 played a pivotal role in stabilizing microtubules for spindle assembly and kinetochore-microtubules attachment. Furthermore, we observed an interaction between KIFC3 and PRC1 during cytokinesis in mouse oocyte meiosis.

### Electronic supplementary material

Below is the link to the electronic supplementary material.

**Supplementary Material 1. Figure S1.** KIFC3 protein expression after KIFC3 morpholino and siRNA injection (A) KIFC3 expression in the KIFC3 siRNA (USA, Santa, sc-146480) injection and control oocytes. Each group contained 150 GV oocytes. Repeat the experiment 3 times. (B) KIFC3 expression in the morpholino (Morpholino oligo: 5’ to 3’ GGCTCAGTAACCTCTTCTGGGTGCC) injection and control oocytes. Each group contained 150 GV oocytes. Repeat the experiment 3 times. (C, D) Using different antibodies to detect KIFC3 expression in the KIFC3 siRNAs injection and control oocytes. siRNAs were designed from Sangon biotech (Shanghai, China). (E) The relative mRNA level of the KIFC3 siRNAs injection and control oocytes. siRNAs were designed from Sangon biotech (Shanghai, China). Each group contained 30 GV oocytes. Repeat the experiment 3 times. (F) PB1 extrusion rate in control and KIFC3 siRNAs injection oocytes. siRNAs were designed from Sangon biotech (Shanghai, China). Each group contained 30 GV oocytes. Repeat the experiment 3 times.

## Data Availability

The supporting data for the conclusions drawn in this research can be accessed within the methods section and/or supplementary materials provided alongside this article.
