# Supplementary figures and images for: Kinesin KIFC3 is essential for microtubule stability and cytokinesis in oocyte meiosis
Source: Cell Commun Signal. 2024 Mar 29;22:199. doi: 10.1186/s12964-024-01589-8 (PMC10979585; doi:10.1186/s12964-024-01589-8)

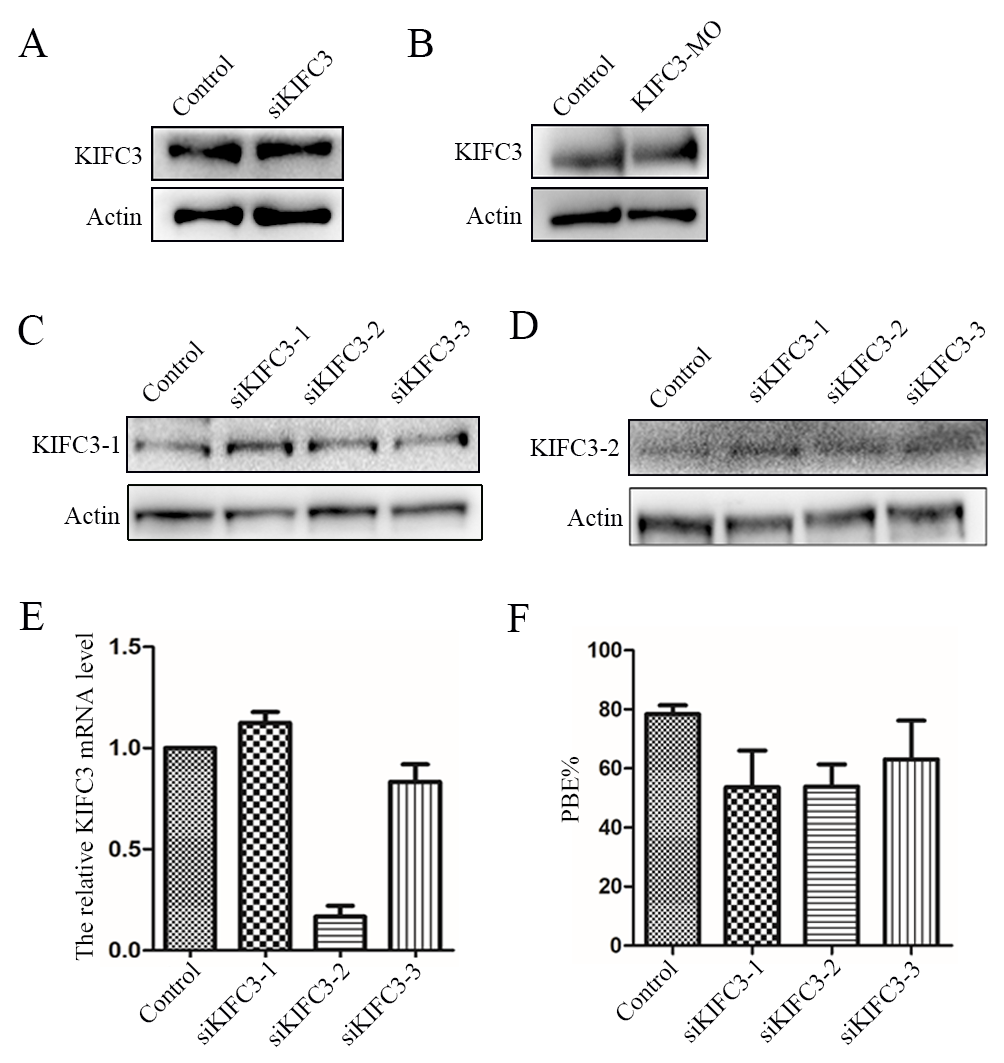

Supplement: Supplementary file 1 — Supplementary Material 1. Figure S1. KIFC3 protein expression after KIFC3 morpholino and siRNA injection (A) KIFC3 expression in the KIFC3 siRNA (USA, Santa, sc-146480) injection and control oocytes. Each group contained 150 GV oocytes. Repeat the experiment 3 times. (B) KIFC3 expression in the morpholino (Morpholino oligo: 5’ to 3’ GGCTCAGTAACCTCTTCTGGGTGCC) injection and control oocytes. Each group contained 150 GV oocytes. Repeat the experiment 3 times. (C, D) Using different antibodies to detect KIFC3 expression in the KIFC3 siRNAs injection and control oocytes. siRNAs were designed from Sangon biotech (Shanghai, China). (E) The relative mRNA level of the KIFC3 siRNAs injection and control oocytes. siRNAs were designed from Sangon biotech (Shanghai, China). Each group contained 30 GV oocytes. Repeat the experiment 3 times. (F) PB1 extrusion rate in control and KIFC3 siRNAs injection oocytes. siRNAs were designed from Sangon biotech (Shanghai, China). Each group contained 30 GV oocytes. Repeat the experiment 3 times. [file 12964_2024_1589_MOESM1_ESM.tif]
